# Single-Crystal to single-crystal copolymerization in a Pt(acac)2•TCNQ cocrystal with new TCNQ reactivity and coordination mode

**DOI:** 10.1063/4.0000999

**Published:** 2025-10-27

**Authors:** Larry R. Falvello, Miguel Baya, Slavomira Šterbinská, Milagros Tomás, Esteban P. Urriolabeitia

**Affiliations:** 1 CSIC-University of Zaragoza, Zaragoza, Aragón, Spain; 2 Aragón Nanoscience and Materials Institute, Zaragoza, Aragón, Spain; 3 Dept. of Inorganic Chemistry, Zaragoza, Aragón, Spain; 4 Instituto de Síntesis Química y Catálisis Homogénea, CSIC-University of Zaragoza, Zaragoza, Aragón, Spain

## Abstract

We report new results involving an unprecedented single-crystal to single-crystal reaction in charge transfer cocrystals of neutral TCNQ with a neutral planar coordination compound of Pt(II).

TCNQ (7,7,8,8-tetracyanoquinodimethane, C_12_H_4_N_4_) is a flat, easily reduced molecule that can form cocrystals exhibiting charge transfer between the TCNQ and its coformer.

A simple Pt coordination compound [Pt(II)(acac)2] (acac is acetylacetonate, C H O ^-^) forms a cocrystal5with7 2 neutral TCNQ. Some forms of stimulus provoke a solid-state oxidative addition reaction in the cocrystal, in which an organometallic copolymer is formed, catena-[Pt(IV)(acac)2-μ(TCNQ2-)]n. The prominent feature of the structure of the [Pt(acac)2]•TCNQ cocrystal, **1**, Fig. 1, is a column of alternating coplanar molecules of the two components, with a slight tilt between the propagation direction of the column and the direction perpendicular to the plane of the molecules.

Following the single-crystal to single-crystal reaction to form **2**, Fig. 2, the columns of independent molecules are replaced by a one-dimensional copolymer in which each Pt center has undergone Pt-C bond formation. The two Pt-C bonds are trans to each other, and the four- coordinate Pt(II) center of [Pt(acac)2] has been oxidized to Pt(IV) and is six-coordinate in the polymer. Each TCNQ is coordinated to two different Pt centers through what were the exocyclic sp2-hybridized C7 and C8 of the original TCNQ. These C atoms are sp3 hybridized in the copolymer, breaking the planarity of the TCNQ, Fig. 2. TCNQ^(2-)^, now dianionic, presents a new coordination mode. This and other features of the reaction and the product will be discussed.

